# Trace Elements, PPARs, and Metabolic Syndrome

**DOI:** 10.3390/ijms21072612

**Published:** 2020-04-09

**Authors:** Yujie Shi, Yixin Zou, Ziyue Shen, Yonghong Xiong, Wenxiang Zhang, Chang Liu, Siyu Chen

**Affiliations:** State Key Laboratory of Natural Medicines and School of Life Science and Technology, China Pharmaceutical University, Nanjing 211198, China

**Keywords:** trace elements, peroxisome proliferator-activated receptors, metabolic syndrome, iron, zinc, copper, selenium

## Abstract

Metabolic syndrome (MetS) is a constellation of metabolic derangements, including central obesity, insulin resistance, hypertension, glucose intolerance, and dyslipidemia. The pathogenesis of MetS has been intensively studied, and now many factors are recognized to contribute to the development of MetS. Among these, trace elements influence the structure of proteins, enzymes, and complex carbohydrates, and thus an imbalance in trace elements is an independent risk factor for MetS. The molecular link between trace elements and metabolic homeostasis has been established, and peroxisome proliferator-activated receptors (PPARs) have appeared as key regulators bridging these two elements. This is because on one hand, PPARs are actively involved in various metabolic processes, such as abdominal adiposity and insulin sensitivity, and on the other hand, PPARs sensitively respond to changes in trace elements. For example, an iron overload attenuates hepatic mRNA expression of *Ppar-α*; zinc supplementation is considered to recover the DNA-binding activity of PPAR-α, which is impaired in steatotic mouse liver; selenium administration downregulates mRNA expression of *Ppar-γ*, thereby improving lipid metabolism and oxidative status in the liver of high-fat diet (HFD)-fed mice. More importantly, PPARs’ expression and activity are under the control of the circadian clock and show a robust 24 h rhythmicity, which might be the reasons for the side effects and the clinical limitations of trace elements targeting PPARs. Taken together, understanding the casual relationships among trace elements, PPARs’ actions, and the pathogenesis of MetS is of great importance. Further studies are required to explore the chronopharmacological effects of trace elements on the diurnal oscillation of PPARs and the consequent development of MetS.

## 1. Introduction

Metabolic syndrome (MetS) is a highly prevalent clinical entity which has become a global epidemic [1]. It increases the incidences of Type 2 diabetes (T2D) and cardiovascular diseases (CVD), representing a great threat to public health and to entire social economies [2]. Among subjects aged 15 years and older, the pooled prevalence is 24.5% in Mainland China [3]. MetS often occurs in populations characterized by excessive nutritional intake and physical inactivity [4]. Metabolic and genetic susceptibility are also potential key risk factors for MetS [5,6]. Abdominal fat accumulation, upregulation of serum triglycerides and glucose, hypertension, and a dysregulated ratio of low-to-high-density lipoprotein levels are the most common features of MetS [7]. Patients exhibiting three or more conditions among obesity, atherosclerotic dyslipidemia, hypertension, hyperglycemia, and aggravated inflammation can be clinically diagnosed as having MetS [8]. In this population, individuals with obvious upper-body obesity are more susceptive to MetS [9].

Previous hypotheses suggested that MetS is initiated by insulin resistance (IR) [10]. There is no doubt that IR causes hyperglycemia, but whether it acts on other metabolic factors is still uncertain [11]. Another possibility is that obesity (or energy imbalance) may be the main cause, due to the close relationship between obesity and all metabolic factors [12,13]. Obesity is an effective clinical indicator of an over-nourished state, but it is not necessarily true that the excessive accumulation of adipose tissue is the real cause of MetS [11]. For example, caloric restriction reverses most metabolic risk factors even in cases of continuous obesity [14]; this fact suggests that a positive energy imbalance (over-nutrition) takes precedence over excessive adipose tissue, which is the main cause of the syndrome [14].

Interest in essential trace and mineral elements has been increasing in recent years [15]. Certain essential trace elements (such as iron, zinc, selenium) play essential roles in maintaining human metabolic homeostasis [16,17,18]. Imbalances in trace elements significantly disrupt energy metabolism, which causes digestive [19], cardiovascular [20], hematological [21], and endocrine diseases [22]. For example, chromium, copper, zinc, and selenium play indispensable roles in cardiovascular protection and cholesterol modulation [23,24]. Specifically, several trace elements, such as zinc, serve as essential components of various enzyme systems, especially, DNA polymerase, glutamate dehydrogenase, lactic dehydrogenase [25]. Therefore, levels of trace and mineral elements are correlated with the occurrence of MetS.

## 2. Trace Elements and Metabolic Syndrome

Trace elements, such as iron, zinc, copper, chromium, selenium, and so on, are micronutrients involved in hundreds of biological processes, including inflammation [26], oxidative stress [27], and lipid metabolism [28]. These elements account for a low percentage of total body weight (less than 0.01%) [29]. Trace elements are widely distributed in nature, as well as in the human body (Table 1). Trace elements can be obtained from food and from the environment; iron, copper, zinc, selenium, cobalt, phosphorus, potassium, and fluorine can come from food; strontium, barium, iodine, and bromine from drinking water [30,31,32].

There are 20 trace elements essential to the maintenance of human physiological homeostasis [33]. They are indispensable substances in cellular and tissue metabolism and play important roles in maintaining healthy physiological states. For example, zinc and iodine act as essential components of DNA polymerase [34] and thyroid hormone [35], respectively; heme iron binds to porphyrin of heme, which combines with oxygen [36]; cobalt performs its physiological functions mainly in vitamin B12, also known as cobalamin, a unique vitamin that contains a metallic element [37]. Trace elements are involved in the various stages of body development, including tissue generation, growth, physiological metabolism, and enhancement of the immune system [30]. Notably, selenium and zinc are even related to prostate cancer and male fertility [38,39]. In 1990, the WHO divided trace elements into three groups: (1) essential elements, including iron, zinc, copper, selenium, iodine, molybdenum, chromium, and cobalt; (2) potentially essential elements, including manganese, silicon, boron, vanadium, and nickel; and (3) potentially toxic elements that are nevertheless essential at low concentrations, including fluorine, bromine, plumbum, cadmium, mercury, arsenic, aluminum, and stannum [40].

**Table 1:** Amount in and recommended daily intake of trace elements for humans.

Elements	Content in the Human Body	RDI	Reference
iron (Fe)	3000–5000 mg	15 mg for man 20 mg for woman	[41,42]
zinc (Zn)	2500 mg	15 mg for man 11.5 mg for woman	[43]
copper (Cu)	100–150 mg	2 mg	[44]
selenium (Se)	14–21 mg	50 μg	[45]
iodine (I)	20–50 mg	150 μg	[46]
molybdenum (Mo)	9 mg	0.1–0.5 mg	[47,48]
chromium (Cr)	6 mg	50 μg	[49]
cobalt (Co)	1.1–1.5 mg	5–45 μg	[50]
manganese (Mn)	12–20 mg	2.5–7 mg	[51]
silicon (Si)	2000–3000 mg	20–50 mg	[52]
boron (B)	50 mg	2–20 mg	[53,54]
vanadium(V)	25 mg	0.1–0.3 mg	[55]
nickel (Ni)	6–10 mg	0.3 mg	[56]
fluorine (F)	2000–3000 mg	0.5–1.0 mg	[52]
bromine (Br)	200 mg	1 mg	[40,57]
plumbum (Pb)	<10 μg/dL Blood	<0.1 mg	[58,59]
cadmium (Cd)	<1 mg/dL Blood	<70 μg	[60]
mercury (Hg)	<0.8 μg/dL Blood	<0.01 mg	[59,61]
arsenic (As)	<1 μg/dL Blood	1 mg	[62]
aluminum (Al)	50–100 mg	1.8–8.4 mg	[63,64]
stannum (Sn)	0.38 mg/dL Blood	0.2–3.5 mg	[65]

RDI, recommended daily intake.

**Figure 1:**
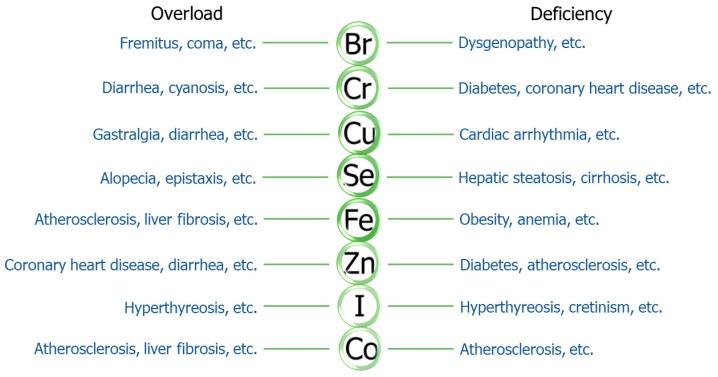
Trace elements and diseases. Overload and deficiency of multiple trace elements are closely correlated to the pathogenesis of numerous diseases. Br, Bromine; Cr, Chromium; Cu, Copper; Se, Selenium; Fe, Iron; Zn, Zinc; I, Iodine; Co, Cobalt.

Both deficiency and overload of trace elements can negatively affect systemic homeostasis. The recommended daily intake (RDI) of different elements varies significantly. For example, the optimal intake range of selenium is 50–200 μg/d [45], while those of copper and fluorine are 2 mg and 0.5–1.0 mg/d [44,52], respectively. Iron overload has been reported to accelerate the process of fatty liver disease [66], liver fibrosis [67], and hepatoma [68] and to highly elevate the risk of cardiovascular events via excessive redox production [69]. On the other hand, deficiency of iron disrupts the normal weight gain and easily drives the process of obesity [70]. Zinc directly regulates the synthesis, storage, and release of insulin; its depletion always causes insulin dysfunctions, ultimately enhancing systemic IR and impairing glucose tolerance [71]. In diabetic patients, zinc supplementation surprisingly improves insulin sensitivity and atherosclerosis [72,73]. Chromium also shows similar effects on insulin and glucose metabolism [74]. Cobalt serves as a component of vitamin B12 [75] and enhances organic iron storage and the absorption of iron and zinc by the intestine [76,77,78]. Molybdenum and fluorine have also been reported to have similar functions in absorbing and utilizing iron [79,80,81], maintaining cardiac energy, and preventing CVD [82]. Studies about copper and manganese have mainly focused on cardiovascular diseases. High serum copper and manganese contents have been reported to be independent risk factors and biomarkers of CVD (e.g., cardiac arrhythmia) in both case–control and large prospective population studies [83]. In contrast, insufficient silicon intake is associated with a high risk of cardiovascular events and increases the case fatality rate [84]. Selenium often acts as a redox scavenger in the human body [27], an action which is essential to the restoration of impaired islets and vascular tissues in diabetes [85,86]. The liver is one of the organs with the highest selenium concentration [87]. Clinical data suggest that patients suffering from chronic liver diseases such as hepatic steatosis and cirrhosis have much lower concentrations of plasma selenium [88]. Chronic selenium supplementation recovers hepatic dysfunction; selenium is thus described as a hepatic protective factor [89]. Regarding non-metallic elements, it has been found that bromide levels are negatively related to levels of triglyceride (TG), cholesterol, and high-density lipoprotein cholesterol (HDL-C) in humans and rats [90]. An in vitro study also illustrated beneficial effects of bromide in hepatocytes recovering from a lipid metabolism disorder [91]. Moreover, an adequate and reasonable intake of boron, which mainly exists in vegetables and fruits, effectively reverses the plasma contents of elevated blood glucose and reduces TG in postmenopausal women [92]. In conclusion, trace and mineral elements are closely correlated to the pathogenesis of metabolic diseases and systemic disorders (Figure 1).

## 3. Peroxisome Proliferator-Activated Receptors Mediate the Effects of Trace Elements on Metabolic Syndrome

**Figure 2:**
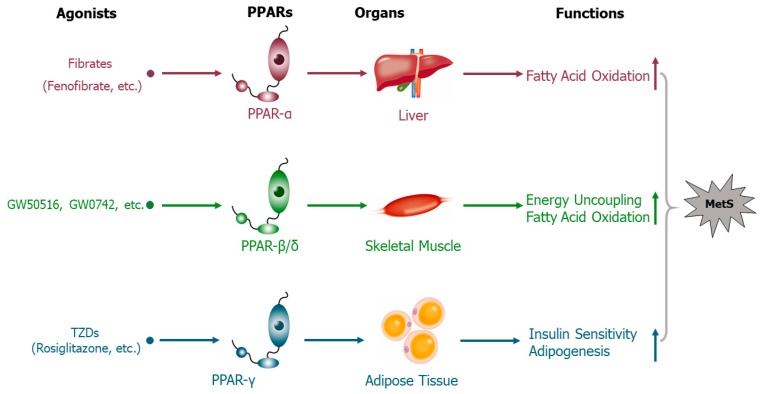
Major roles of peroxisome proliferator-activated receptor (PPAR) isotypes in metabolic syndrome (MetS). PPARs are a class of nuclear transcriptional factors activated by physiological stimuli (fatty acids and eicosanoids) and synthetic small molecules (fibrates for PPAR-α; GW501516, GW0742, bezafibrate, and telmisartan for PPAR-β/δ; thiazolidinediones (TZDs) for PPAR-γ). PPAR-α is mainly expressed in liver tissue, where it controls a set of genes facilitating fatty acid oxidation, thereby lowering circulating triglyceride levels. PPAR-β/δ modulates a series of genes involved in energy uncoupling and fatty acid oxidation in skeletal muscle, resulting in increased energy expenditure and reduced plasma triglyceride levels. PPAR-γ is abundantly expressed during increasing adipogenesis in adipose tissues, where it governs multiple genes and thereby improves insulin sensitivity and decreases lipolysis. All the members of the PPAR family can be activated by relevant agonists. Although different PPARs have unique non-overlapping patterns of biological functions, all three isoforms act on given tissues and share similar biological functions.

Peroxisome proliferator-activated receptors (PPARs) are members of the nuclear receptor superfamily and include three nuclear receptor isoforms which are ligand-inducible transcription factors, i.e., PPAR-α, PPAR-β/δ, and PPAR-γ [93]. Through heterodimerizing with the retinoid X receptors (RXRs), which then bind to PPAR-responsive regulatory elements (PPRE), PPARs control a group of genes involved in energy homeostasis, insulin sensitivity, lipid metabolism, and maintenance of metabolic homeostasis [94,95]. The first identified member of the PPAR group, PPAR-α, is highly expressed in highly energy-demanding tissues which show high rates of β-oxidation, such as the liver, kidney, heart, and skeletal muscle [96]. Activation of PPAR-α mainly occurs under energy deprivation [96]. It has been reported that PPAR-α mediates the hypolipidemic function of fibrates (selective PPAR-α agonists) in the treatment of hypertriglyceridemia, being the star regulator of lipid metabolism [97]. Specifically, an animal study revealed that oral administration of fenofibrate effectively lowered the serum TG levels of obese mice through upregulating hepatic very low density lipoprotein receptor (VLDLR) [98]. PPAR-β/δ is the least well-characterized isotype of the PPAR family. It is mainly expressed in skeletal muscle and acts as a key regulator of muscular lipid balance [99]. Studies of systemic PPAR-β/δ agonists (GW501516, GW0742, bezafibrate, telmisartan, etc.) have demonstrated the pivotal role of this gene in lipid metabolism. For example, researchers noted the fact that GW501516 treatment improved MetS in obese monkeys and mice. Moreover, in GW0742-treated L6 rat myocytes, fatty acid uptake and β-oxidation were robustly increased compared to a control group [100,101]. These data were also confirmed in PPAR-β/δ-overexpressing activated mouse C2C12 myoblasts [102]. However, so far there have been no clinical studies to support the findings in animal models. PPAR-γ serves as an important mediator in energy balance and cell differentiation. IR is the central node of metabolic syndrome, and impaired PPAR-γ function is reported to induce severe IR in mouse adipose tissues [103]. Conversely, PPAR-γ positively adjusts glucose metabolism by increasing the insulin sensitivity of peripheral tissues, thus improving IR. Thiazolidinediones (TZDs), synthetic insulin-sensitizing PPAR-γ agonists, like rosiglitazone, pioglitazone, and troglitazone, effectively improve IR and lower the blood glucose of T2D patients and have been widely prescribed to treat T2D in the clinic [104]. Consistent with other anti-diabetic strategies (oral anti-diabetic agents and insulin), treatment-related excessive weight gain and fluid retention are common side effects of TZDs [105]. Besides, a previous population-based study of older patients with T2D demonstrated that TZDs treatment, primarily with rosiglitazone, was associated with a higher risk of and mortality due to cardiovascular events, such as congestive heart failure and acute myocardial infarction [106]. Additionally, it has been reported that activation of PPAR-γ accelerates and elevates lipolysis in rat peripheral tissues (especially in adipose tissue) [107] and that this is followed by upregulation of hormone-sensitive lipase (*Hsl*) mRNA expression in rat hepatocytes and preadipocytes [108]. PPARs are obviously correlated with the pathogenesis of various metabolic diseases, such as T2D [109], obesity [110], non-alcoholic fatty liver disease (NAFLD) [111], and atherosclerosis [112]. The major roles and functions of the PPAR isotypes are depicted in Figure 2. Studies on the functions of trace elements in MetS have attracted growing interest. Among these studies, PPARs have been noted to be directly or indirectly modulated by trace elements in different organs and tissues (Table 2). Thus, PPARs may serve as crucial mediators of trace elements under MetS.

### 3.1. Iron

Iron is extensively distributed throughout the whole human body and shows the highest content among all trace elements found in humans [146]. It is an essential mineral required for a variety of molecules to maintain their normal structures and functions in growth and proliferation. Iron is distributed in almost all organs, especially the liver, spleen, and lungs [147]. Iron exists in the human body in two main forms: heme and non-heme [148]. The heme form includes hemoglobin, myoglobin, cytochrome, and various enzymes, while the non-heme form includes ferritin, lactoferrin, hemosiderin [149,150]. Importantly, 60%–70% of iron in the body exists in the form of hemoglobin [36].

Iron uptake occurs mainly through food intake, and the mineral is easily absorbed throughout the whole gastrointestinal tract [151]. Iron overload is always manifested as a gross elevation in serum iron and hepatic iron storage [152]. In daily life, a high dietary intake of iron through meat or nutritional supplements is a potential cause of iron overload [153]. Hepatic iron overload can be found in numerous chronic liver diseases [152]. Some animal and epidemiological studies have suggested that high iron levels may have a harmful impact on glucose and lipid metabolism [154,155,156]. It is noteworthy that iron overload attenuates the hepatic expression of *Ppar-α*, which is an important transcriptional factor that promotes lipid and lipoprotein metabolism [113]. Bonomo et al. [113] reported evidence that iron is involved in the pathogenesis of non-alcoholic steatohepatitis (NASH). Their data showed that intraperitoneal injection of iron dextran, when associated with a high-fat diet (HFD), caused increased serum cholesterol levels due to a reduction in *Ppar-α* mRNA expression in the liver tissue of hamsters. So, decreased *Ppar-α* expression might be an important mechanism underlying the iron overload-mediated disruption of lipid metabolism.

Hepatic fibrosis is an exacerbated wound-healing response with excessive synthesis and deposition of extracellular matrix (ECM) in the liver [157]. The ECM components are synthesized by hepatic stellate cells (HSC) [158]. For this reason, excessive HSC activation is believed to be the main cause of the hepatic fibrotic process and maintenance. Gardi et al. [159] demonstrated that a 48 h incubation of a solution of ferric chloride and citrate abnormally stimulated rat HSCs, and iron chelators remarkably reversed the activation, upregulated pro-apoptotic proteins, and therefore reduced fibrosis. Various in vitro studies have reported that iron treatment activated HSCs, which was accompanied by decreasing *PPAR-γ* expression. Dias et al. [160] discovered the fact that fructose-1,6-bisphosphate (FBP), serving as a novel iron chelator, could reverse activation in the mouse GRX HSC cell line, leading to a quiescent state, by recovering *Ppar-γ* expression dampened by iron.

It is well recognized that a temporal iron deficiency sensitizes insulin action [161], but chronic iron deficiency can accelerate the development of cardiovascular diseases [162]. Minamiyama et al. [114] concluded that the expression of PPAR subtypes in diabetic rats was influenced by iron levels in the liver and pancreas. In particular, mRNA expression of *Ppar-α* and *Ppar-γ* in iron-deficient rats was decreased in the pancreas but was not altered in the liver. Another member of the PPAR family, *Ppar-β/δ*, showed elevated mRNA levels and maintained this tendency in both liver and pancreas upon iron depletion.

### 3.2. Zinc

Zinc is an essential trace element and micronutrient and plays a vital role in various physiological processes. Human nutritional requirements for zinc are second to iron [163]. Its deficiency is remarkably associated with inductive oxidative stress [164], inflammatory events [165], and vascular dysfunction [166]. Epidemiological studies suggest that low serum levels of zinc are inversely associated with multiple diseases, such as diabetes [167], coronary artery disease [168], and Parkinson’s disease [169].

Zinc plays both catalytic and structural roles in nearly 300 specific enzymes and thousands of “zinc finger” protein domains, through which zinc also plays regulatory functions in cellular signaling pathways [170,171]. Coincidentally, the DNA-binding domain (DBD) of PPAR, PPRE, contains two classic “zinc fingers” [172], meaning that zinc may be a critical component of gene expression and regulation by PPARs. Hence, depletion of zinc may partially impair the transcriptional function of PPAR complexes.

As the largest human metabolic organ, the liver plays a crucial role in maintaining systemic zinc homeostasis. Plenty of chronic hepatic metabolic abnormalities, including IR [173], NAFLD [174], hepatic steatosis [173], liver cirrhosis [175], and hepatic encephalopathy [176], are often ascribed to systemic zinc depletion. In the livers of hepatic steatosis mice, zinc has been considered to be closely related to the DNA-binding activity of PPAR-α [117]. Therefore, zinc deficiency may result in a decline of PPAR-α function, thereby facilitating a detrimental alteration of lipid peroxidation, ultimately exacerbating hepatic steatosis [177]. Sugino et al. [178] investigated the effect of zinc (polaprezinc) in a NASH mouse model. Zinc supplementation did not affect the steatosis but, surprisingly, attenuated fibrosis in the liver. Another study reported that treatment with zinc sulfate reversed alcohol-induced steatosis in male mice via reactivation and recovery of hepatocyte nuclear factor-4α (*Hnf-4α*) and *Ppar-α* [117]. Combined with more clinic reports, we may conclude that zinc supplementation could therefore be considered as an optional treatment for patients suffering from some specific chronic liver diseases [176,179,180,181].

Endothelial cell dysfunction and activation play major roles in the development and progression of CVD [182]. Zinc has shown extensive and potent antioxidant and anti-inflammatory properties [183]. Zinc deficiency evokes oxidative stress and negatively affects endothelial cell function [164]. Shen et al. and Meerarani et al. [116,184] demonstrated that insufficiency of zinc-induced vascular pro-inflammatory parameters was associated with dampened NF-κB and PPAR signaling in mice and porcine endothelial cells, respectively. Their original research supports the concept that adequate zinc supplementation could reverse impaired anti-inflammatory and protective functions of PPARs (PPAR-α and PPAR-γ) in endothelial cells.

### 3.3. Copper

Copper is an essential element for most living organisms and plays an important role in physiological processes [185]. Needed in only trace amounts, the human body contains approximately 100 mg of copper [186]. Insufficient intake of copper often leads to anemia, paratrichosis, infertility, and brain disorders [187]. There is also no doubt that copper is toxic at high levels, although it is an essential micronutrient for human bodily functions [188]. An overload of copper quickly results in a detrimental alteration of living organisms, through liver cirrhosis, emesis, diarrhea, arthritis, cognitive decline, and cardiac arrhythmia [187]. Thus, it is urgent and vital to consider balanced homeostatic mechanisms of copper intake, absorption, and excretion. Normally, copper intake comes from various foodstuffs, such as milk, meat, seafood, vegetables, and fruits, which are all rich in copper [189]. Fifteen minutes after dietary copper enters the human body, copper is absorbed into the blood and erythrocytes [190]. It plays essential roles in catalyzing and activating the production of ferroheme and the absorption and utilization of iron through collaboration with transferrin [191]. Copper generally exists in tissues in the form of organic compounds, most of which are metalloproteins. Metalloproteins normally act as enzymes catalyzing electron transfer and oxidation–reduction reactions; these enzymes include tyrosinase, monoamine oxidase, peroxidases, superoxide dismutase, and hemocyanin [192].

Fat is the largest energy reserve in mammals [193]. Most tissues are involved in fatty acid metabolism, but three are quantitatively more important than others: adipose tissue, skeletal muscle, and liver tissue. One study in rabbits by Liu et al. [28] showed evidence that addition of extra dietary copper decreased hepatic fat content, reduced intramuscular fat accretion, and promoted skeletal muscle growth presumably through activating PPAR-α signaling in liver, adipose tissue. and skeletal muscle. As mentioned above, acute exposure to high amounts of copper deteriorates tissue function. Reports on aquatic *Takifugu fasciatus* illustrated the fact that copper sulfate accumulation and stress disrupted aquatic lipid homeostasis in the liver, which was accompanied by upregulated *ppar-γ* [119,120].

### 3.4. Selenium

Selenium is incorporated into selenoproteins, which have a wide range of pleiotropic effects, ranging from immune-enhancing, antioxidant, and anti-inflammatory effects to the production of active thyroid hormone [194]. Human beings absorb selenium only through the duodenum, not the stomach or any other section of the intestinal tract [195]. This is the reason why humans are generally lacking in selenium. In contrast to many other micronutrients, the intake of selenium varies hugely worldwide, ranging from deficiency to toxic concentrations that cause garlic breath, hair and nail loss, disorders of the nervous system, poor dental health, and paralysis [196]. The recommended dietary selenium intake ranges from 7 to 4990 μg per day worldwide, with mean values of 60 μg per day in China and 40 μg per day in Europe [194].

In the livers of HFD-induced NAFLD rats, selenium supplementation recovered dyslipidemia and improved liver function and hepatic steatosis by activating *Ppar-α* expression and subsequently elevating fatty acid oxidation [126]. Selenium-enriched probiotics have been confirmed to have a great effect in improving lipid metabolism, antioxidative status, and histopathological lesions in HFD-fed mice. Among genes whose expression was altered in the liver, *Ppar-α* was upregulated [126]. IR plays a pivotal role in the pathogenesis of NALFD in the setting of IR syndrome or MetS [197]. In a previous study, selenium-enriched green tea Ziyang reduced IR, together with oxidative stress and hepatic steatosis, in high-fructose-fed mice [198]. Mueller et al. [199] also reported a similar effect of selenium in the form of selenate, but not selenite; its administration in *db/db* diabetic mice improved IR syndrome by increasing the expression of *Ppar-γ* and reducing the activity of liver cytosolic tyrosine phosphatases.

PPAR-γ, as a transcriptional node, participates in an important signaling pathway that occurs at the intersection of depression and obesity. Donma et al. [200] combined the epidemiological evidence that selenium supplementation alleviates inflammatory signaling pathways and inflammatory cytokines, e.g., TNF-α, IL-1β, and prostaglandin E2 (PGE2) and interacts with various stages relevant to depression, the so-called obesity-associated parameters. They suggested that lipophilic selenium compound supplementation and fortification could be employed as a novel PPAR-γ agonist to alleviate obesity as well as depression.

Selenium depletion significantly heightens the risk of cardiovascular diseases by reducing the concentration and activity of selenoproteins that act as predictors of cardiovascular events [194]. Clinically, the administration of selenium to patients with cardiomyopathy improves an extensive range of cardiac functions [201]. Selenium activates myocardial calcium and ATPase, thereby recovering myocardial contractility [202]. Evidence has confirmed that most heart disease patients show much lower selenium levels than healthy cohorts in their blood and heart [203]. Nowadays, supplementing selenium is becoming an important strategy for preventing cardiopathy [204]. Recently, researchers used thiamine (vitamin B1) and sodium selenite to accelerate and reverse the basal transcriptional activity of *Ppar-γ*, which was impaired by citreoviridin (a mycotoxin, ATP synthase inhibitor, and one of the etiological factors of cardiac beriberi and Keshan disease) in mouse heart and H9c2 cardiomyocytes [127]. A recent clinical study reported the effects of selenium supplementation on the elevated gene expression of *Ppar-γ* in the lymphocytes of women with polycystic ovary syndrome (PCOS), who were candidates for in vitro fertilization (IVF) [124]. An animal experiment in chicken pancreas illustrated the antagonistic effect of sodium selenite on cadmium-induced apoptosis, which involved the recovering of the impaired PPAR-γ/PI3K/Akt pathway by cadmium [121]. Besides, selenium-enriched probiotics (*Lactobacillus acidophilus* and *Saccharomyces cerevisiae*) have been reported to repress the gene expression of *Ppar-γ*, thereby improving lipid metabolism, in HFD-fed mice [126]. Conflicting results have emerged when comparing PPAR-γ modulation by selenium in various studies, whose reason may be that various sources of selenium (selenite and selenium-enriched probiotics) and diverse target organs/tissues (heart, liver, pancreas, and immune system) were considered in these studies.

### 3.5. Other Essential Trace Elements

There are four other microelements (iodine, molybdenum, chromium, and cobalt) essential for human function, besides the aforementioned iron, zinc, copper, and selenium. It has been widely recognized that iodine has a series of beneficial physiological functions [205]. Iodine deficiency disorders are among the biggest public health problems worldwide today, with populations in southern Asia, Latin America, and Sub-Saharan Africa particularly affected [206]. It is noteworthy that insufficient iodine intake in adults results in a high risk of multiple cancers (goiter, mammary cancer); iodine supplementation has been considered as an adjuvant therapy for these cancers [129,207]. Research by Aceves et al. [128] and Alfaro et al. [129] suggested the participation of PPARs in the antineoplastic effect of iodine; I_2_ in drinking water effectively dampened the expression of *Ppar-α* and elevated that of *Ppar-γ* in tumoral mammary glands of rats. Similarly, chromium, which has a concentration of only 6–7 mg in the human body, serves as a necessary regulator of normal body weight [208] and blood glucose level [208] and for cardioprotection [209]. Chromium picolinate significantly induces *Ppar-δ* mRNA expression in skeletal muscle of HFD-fed rats [131], as PPAR-δ is a well-known modulator of fatty acid metabolism in skeletal muscle. Chen and colleagues demonstrated that oral chromium moderately improved impaired *Ppar-α* in the liver of HFD-fed mice [133]. Coincidently, in Type 2 diabetic rats, a safe dose of malate acid chromium activates *Ppar-γ* to exert its hypoglycemic effect [132]. As for cobalt, limited studies have revealed that cobalt chloride, a chemical hypoxia mimetic, reduces mRNA levels of *PPAR-α* and *-γ* in the heart and in Caco-2 cells [135,210].

Over the years, some trace elements which may exert potential toxicity but have essential effects at low concentrations in humans have been recognized. In our previous study, we focused on bromine and we found that sodium bromide alleviated excessive lipid accumulation and recovered lipid dysfunction by activating PPAR-α signaling in mouse primary hepatocytes, since PPAR-α is a key participant in the induction of fatty acid oxidation [91]. As a non-metal trace element, chronic exposure to inorganic arsenic (arsenic trioxide) strongly impaired *Ppar-γ* expression in the liver and in the 3T3-L1 cell line [211].

## 4. Trace Elements Supplementation and Perspectives

Nowadays, in addition to environmental resources, we widely supply the trace elements through healthcare agents or additives. The first generation of micronutrient additives was produced decades ago and mainly included sulfates and oxides, while the second generation, which has recently begun, mostly includes organic salts such as zinc gluconate, zinc citrate, iron lactate, and iron gluconate [212]. Given that the biological activities of trace elements are controversial under different settings, we speculated that these controversial effects are partially caused by the circadian clock, which orchestrates the biological processes in a 24 h cycle during a day [94]. Moreover, modern chronotherapeutics, which refers to the combination of systemic diurnal activity and clinical therapy, explores the optimal time of medication in a clinical context to separate drug efficacy from toxicity, thereby achieving the purpose of increasing the efficacy and tolerance of drugs [213]. Thus, chronotherapy is now of great importance to minimize these controversial effects. However, currently, there is no convincing information on the usefulness of compounds containing trace elements. Hence, chronotherapeutics may maximize the effects of trace elements on MetS, as well as minimize their potential side effects. All functions in humans are highly organized in time as biological rhythms of diverse periods, both in health and in disease. It is well-known that the biological rhythms significantly affect the responses of patients to diagnostic tests, and rhythmicity in the pathophysiology of disease is a basis for chronotherapeutics [214]. As healthcare agents or additives, trace element intake has the potential to develop “chronotherapeutic pharmacological properties”. Given that the ions of trace elements may pass cytoplasmic membranes through certain ionic channels or receptors [146,215,216,217] and the fact that the expression of multiple ionic channels and receptors exhibits diurnal regulation [217,218,219], we suggest that the absorption of these micronutrients via channels and receptors also shows a circadian pattern. Besides, all three PPAR isoforms were found to be rhythmically expressed in some mouse tissues [220]. Among these, PPAR-α and PPAR-γ are direct regulators of the core clock components BMAL1 and REV-ERBα. Conversely, PPAR-α is also a direct target of BMAL1 [94]. In the context of chronotherapeutics, the rhythms of potential targets, especially PPARs, should be considered and compared.

## 5. Conclusions

This review summarizes the current knowledge on various potential trace elements that modulate PPARs expression and activity. PPARs, members of the nuclear receptor superfamily and transcriptional factors, may serve as effective molecular targets of trace elements in the treatment of MetS. Since the nuclear location and epigenetic modification of PPARs play mainstream roles in their transcriptional function [221,222], it is worthwhile to persistently explore the mechanisms by which trace elements may influence the subcellular location and epigenetic modification of PPARs.

## Figures and Tables

**Table 2:** Effects of trace elements on PPARs modulation in multiple diseases and models.

Elements	Diseases or Models	Organ or Cells	Doses of Elements	Change of PPARs
Fe	Hyperlipidemia, Hamsters	Liver	10 mg/d *i.p.*	PPAR-α↓ [113]
	Diabetes, Rats	Pancreas	De	PPAR-β/δ↑ [114]
	Oxidative Stress, Rats	Central Nervous System	3 mM	PPAR-γ↑ [26]
Zn	Atherosclerosis	HAECs	15 μM	PPAR-α↑ [115]
	Inflammation	PPAECs	12 μM	PPAR-γ↑ [116]
	Steatosis, Mice	Liver	75 mg/L Liquid Diet	PPAR-α↑ [117]
	Sepsis, Mice	Lung	1.3 mg/kg BW *i.p.*	PPAR-γ↑ [118]
Cu	Rabbits	Liver, Muscle, Adipose Tissue	5–45 mg/kg Diet	PPAR-α↑ [28]
	Pufferfish	Liver	24–98 μg/L Water	PPAR-γ↑ [119,120]
Se	Chicken	Pancreas	2 mg/kg Diet	PPAR-γ↑ [121]
	Infection	Mammary Gland	De	PPAR-γ↓ [122]
	Proliferation	HaCaT Keratinocytes	10 μM	PPAR-β/δ↑ [123]
	PCOS, Human	Lymphocytes	200 μg/d *p.o.*	PPAR-γ↑ [124]
	Diabetes, Human	Macrophages	100–300 μg/d *p.o.*	PPAR-γ↑ [125]
	HFD-fed Mice	Liver	0.3 μg/d Diet	PPAR-α↑, PPAR-γ↓ [126]
	Heart Damage, Mice	Heart, H9c2	9 mg/L Water, 5μM	PPAR-γ↓ [127]
I	Mammary Cancer, Rats	Tumor	0.05% in Water	PPAR-α↓, PPAR-γ↑ [128,129]
Cr	Exercise-trained Rats	Liver, Muscle	4 mg/kg BW *i.g.*	PPAR-γ↑, PPARβ/δ↑ [130,131]
	Diabetes, Rats	Adipose Tissue	80 μg/kg BW *i.g.*	PPAR-γ↑ [132]
	NAFLD, Mice	Liver	80 μg/kg BW *i.g.*	PPAR-α↑ [133]
Co	Hypoxia	Trophoblast Cells	100μM	PPAR-α/β/γ↓ [134]
	Hypoxia, Rats	Heart	60 mg/kg BW *i.p.*	PPAR-α↓ [135]
Mn	Neurotoxicity	U87, SK-N-SH	4 mM	PPAR-α/β/γ↓ [136]
	Oxidative Stress, Mice	Mitochondria	De	PPAR-α↑ [137]
Si	-	-	-	PPAR-α/β/γ↑ [138]
V	Adipogenesis	3T3-L1	2.5–10 μM	PPAR-γ↓ [139,140]
	db/db Mice	Adipose Tissue	0.05 mmol/kg BW *i.g.*	PPAR-γ↑ [141]
Br	Hyperlipidemia	Hepatocytes	1–10 μM	PPAR-α↑ [91]
Cd	Chicken	Pancreas	150 mg/kg Diet	PPAR-γ↓ [121]
Hg	HFD-fed Mice	Adipocytes	1 mg/kg BW *s.c.*	PPAR-α↓, PPARγ↓ [142]
As	-	hMETSCs	0.2–4μM	PPAR-γ↓ [143]
	Adipogenesis	C3H/10T1/2	6 μM	PPAR-γ↓ [144]
	HFD-fed Mice	Liver	3 mg/L Water	PPAR-γ↓ [145]

De, deficiency; BW, body weight; VECs, vascular endothelial cells; PPAECs, porcine pulmonary artery endothelial cells; PCOS, polycystic ovary syndrome; HFD, high-fat diet; NAFLD, non-alcoholic fatty liver disease; hMSCs, human mesenchymal stem cell.
